# Photodegradation of Brilliant Green Dye by a Zinc bioMOF and Crystallographic Visualization of Resulting CO_2_

**DOI:** 10.3390/molecules26134098

**Published:** 2021-07-05

**Authors:** Paula Escamilla, Marta Viciano-Chumillas, Rosaria Bruno, Donatella Armentano, Emilio Pardo, Jesús Ferrando-Soria

**Affiliations:** 1 Departament de Química Inorgànica, Instituto de Ciencia Molecular (ICMOL), Universitat de València, Paterna, 46980 València, Spain; paula.escamilla@uv.es (P.E.); marta.viciano@uv.es (M.V.-C.); 2 Dipartimento di Chimica e Tecnologie Chimiche, Università della Calabria, 87036 Cosenza, Italy; rosaria.bruno@unical.it

**Keywords:** metal-organic frameworks, amino acids-derived ligands, water remediation, photocatalytic degradation, single-crystal X-ray crystallography

## Abstract

We present a novel bio-friendly water-stable Zn-based MOF (**1**), derived from the natural amino acid *L*-serine, which was able to efficiently photodegrade water solutions of brilliant green dye in only 120 min. The total degradation was followed by UV-Vis spectroscopy and further confirmed by single-crystal X-ray crystallography, revealing the presence of CO_2_ within its channels. Reusability studies further demonstrate the structural and performance robustness of **1**.

## 1. Introduction

One of the main challenges that modern society faces is related, undoubtedly, to the contamination of aquatic environments, which is mainly caused by human/industrial activities [1,2]. Among the wide diversity of inorganic/organic chemical pollutants, organic dyes—waste generated in the cosmetic, textile, tannery, or food industries, among others—constitute one of the major contaminants in industrial wastewater [3].

Proposed solutions for the removal of such organic contaminants include precipitation, coagulation/flocculation, membrane technology, or biological processes [4]. However, probably the two most promising technologies for this purpose are based on the straightforward capture of the organic dyes by a porous material or their in-situ photocatalytic degradation [5,6]. In particular, metal-organic frameworks (MOFs) are porous crystalline materials that have already been shown to be efficient in the last two approaches [7,8,9,10,11,12,13].

MOFs [14,15] attract broad attention from many research groups given the great variety of applications they can exhibit [14,15,16,17,18,19,20,21]. The reasons for such a variety of applications are related to a number of unique characteristics such as permanent porosity [22,23], a thrilling host-guest chemistry [24,25]—which can be tuned for fine control over the size, shape, and functionality of MOF channels—and the possibility of using single-crystal X-ray diffraction [26,27,28,29,30,31] (SCXRD) to visualize what is going on within their channels [32].

In particular, MOFs have recently been used in water remediation with exceptional results. For example, the easy of functionalizing, pre- or post-synthetically, MOF channels has led to the preparation of specifically designed MOFs for the selective and efficient capture of both organic and inorganic contaminants [8,33,34,35]. Moreover, certain specifically designed MOFs have already been shown to be effective as photocatalysts to degrade organic pollutants—i.e., organic dyes– into less toxic intermediates or fully degrade them into CO_2_ and H_2_O [10,36,37,38,39,40]. In particular, several Zn(II)-based MOFs have recently been reported, showing, by analogy with zinc oxide based photocatalysts [41,42], moderately good efficiencies as photocatalysts towards different organic dyes [43,44,45].

## 2. Results

**Figure 1:**
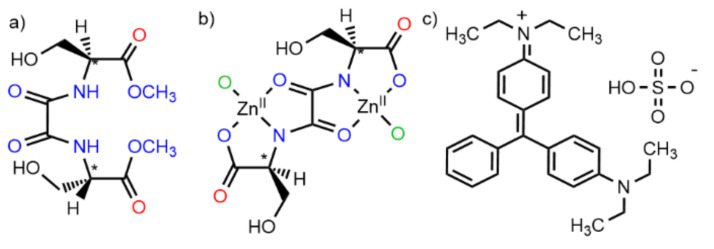
Chemical formulas of the proligand (*S*,*S*)-serimox (**a**) and the secondary building units (SBU) consisting of Zn^II^ dinuclear units (**b**). Red oxygen atoms coordinate Zn^II^ atoms from another SBU, whereas green oxygen atoms belong to neighboring SBUs. (**c**) Chemical formula of brilliant green dye.

In this communication, we report the preparation and total characterization of a novel eco-friendly Zn-based MOF, derived from the natural amino acid *L*-serine, with formula {Zn^II^_2_[(*S*,*S*)-serimox](H_2_O)_2_] ··· H_2_O (**1**) ((*S*,*S*)-serimox = [bis[(*S*)-serine]oxalyl diamide] (Figure 1a,b). The final material is capable of photodegrading brilliant green (BG) dye (Figure 1c) in only 120 min with an efficiency of 100% in the absence of any other oxidant or co-catalyst. In addition, the high robustness and crystallinity of 1 also allowed us to obtain the crystal structure of 1—with the help of SCXRD—after the photocatalytic process, which shows, unambiguously, the presence, within the channels, of CO_2_ molecules resulting from the photodegradation of BG dyes.

**Figure 2:**
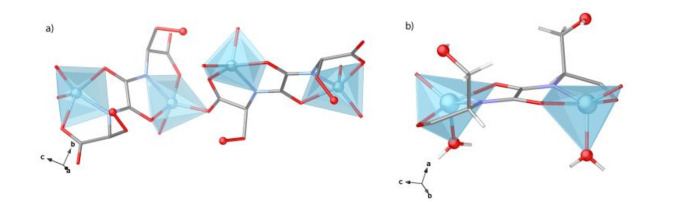
Perspective view of a fragment of 1 emphasizing the central dinuclear Zn_2_ SBU (**b**) unit and their connection to neighboring Zn^2+^ cations (**a**). Color code: Zn, O, and N atoms are represented as cyan, red, and deep blue spheres, respectively, whereas C atoms are depicted as grey sticks.

**Figure 3:**
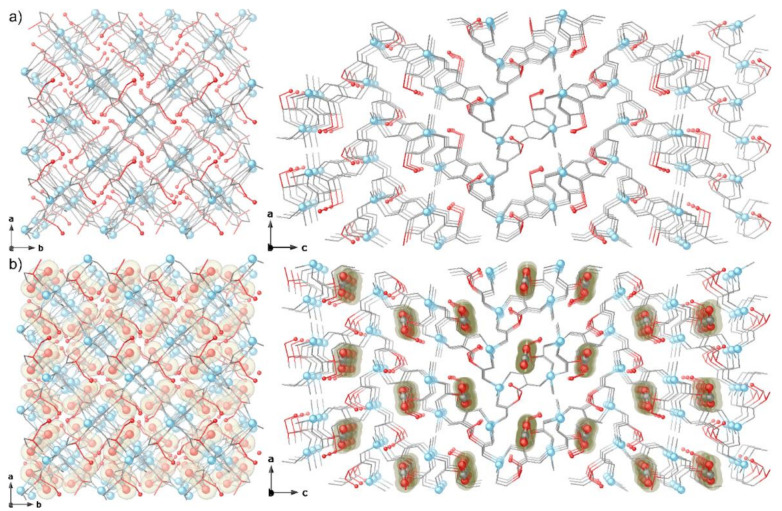
Perspective views of the porous networks of 1 (**a**) and CO_2_@1 (**b**) along the *c* (**left**) and *b* (**right**) axes. Color code: Zn atoms and C and O from guest CO_2_ molecules are represented as cyan, grey, and red spheres, whereas serimox ligands—with the exception of serine residues—are represented by grey sticks. –CH_2_OH groups and N atoms are represented as cyan, red, and deep blue spheres, respectively, whereas C atoms are depicted as grey sticks.

The crystal structure of 1 was first determined at 100 K. It crystallizes in the chiral *P*4_1_2_1_2 space group of the tetragonal system and consists of a chiral 3D pillared square grid where [Zn^II^_2_(*S,S*)-serimox] moieties are located on the vertices of the edges (Figure 2 and Figure 3a). The robust uni-nodal three-connected srs nets are built up from *trans* oxamidato-bridged Zn(II) dimeric units, {Zn^II^_2_[(S,S)-serimox]} (Figure 2 and Figure S1), which are connected to each other through their carboxylate groups (Figure 2, Figures S1 and S2).

Within the {Zn^II^_2_[(S,S)-serimox]} moieties, each Zn(II) metal ion results in a highly distorted pyramidal coordination being linked by nitrogen and oxygen atoms from the serimox ligand [Zn-N 1.985(4)Å and Zn-O_ser_ ranging from 1.958(3) to 2.215(4) Å] and a terminal water molecule [Zn-O_water_ 2.011(4) Å] (Figure 2b). The 3D network features two types of small pore, different in size and shape, propagating along the *b* and *c* axes (Figure 3, Figures S2 and S3). Hydrophilic square sized pores (Figure 3a, left and Figure S3) and hydrophilic irregular pores of medium size (virtual diameters of ca. 0.30 and 0.40 nm, respectively) (Figure 3a, right and Figure S2) are decorated by the primary alcohol group of serine moieties, pointing inwards towards the voids, and accountable for its resulting host–guest chemistry.

Crystals of 1, left in a sealed glass tube containing an aqueous solution of BG dye for one week, after irradiation in the range 250–350 nm for 60 min were analyzed by SCXRD at a temperature of 100 K and the crystal structure of CO_2_@1 was determined, revealing a slight deformation of the framework—likely correlated to CO_2_ adsorption process—but still isomorphous to 1, crystallizing in the *P*4_1_2_1_2 space group. Adsorption of CO_2_ gas—produced by the photocatalytic process at 298 K—results in linear negative expansion. Pores that run parallel to the *c*-axis contract upon CO_2_ inclusion (Δa, Δb slightly < 0; Δc < 0; ΔV < 0). The largest change in dimensions is observed for the *c* axis, accounting for a small decrease in the unit–cell volume (ΔV ≈ 48 Å^3^, Table S1).

**Figure 4:**
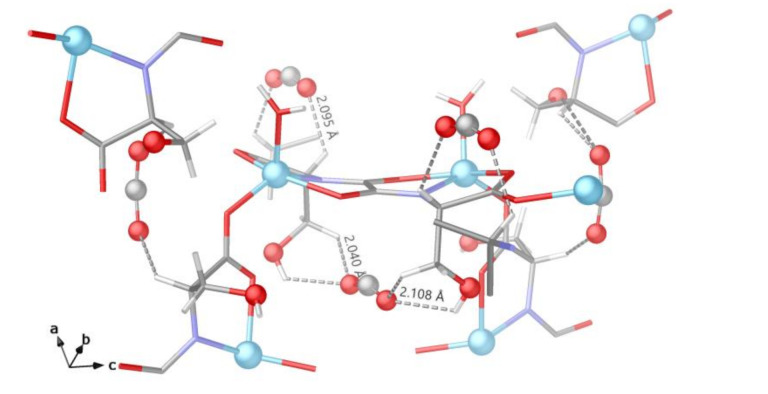
Details of CO_2_@1 crystal structure showing the adsorption sites identified for CO_2_ adsorbed molecules. Color code as in Figure 3.

The crystal structure of the adsorbate CO_2_@1 clearly evidences the presence of CO_2_ guest molecules hosted in the hydrophilic irregular pores of 1 (Figure 3b, Figures S4 and S6. A primary adsorption site can be identified on the CO_2_ molecule (Figure 4), where the plane containing the ligands are situated at only 2.83(1) Å [distance between centroid of oxamate core and C atom of CO_2_ molecule] from the C atom of CO_2_ molecules occupying the primary site, suggesting an interaction between the C(δ^+^) atom of the CO_2_ molecule and the O lone pair of the oxamate core of the ligand, as observed before [46]. The described guest molecule orientation generates a kind of π(CO_2_)···π(oxamate) dimer exhibiting C(oxamate)···O(CO_2_) and C(CO_2_)···O(oxamate) symmetric contacts of 3.08(1) and 3.32(1) Å, respectively. The secondary adsorption sites identified are situated at the center of the pores, where CO_2_ molecules are packed with closest contact with the framework through the H_2_C- and HC- of the ligand [shortest O-C-O····H-C- at 2.04(3) and 2.09(3) Å, for H_2_C- and HC- fragments, respectively] (Figure 4, Figures S4 and S5). The alcoholic fragment makes a contribution as well, being situated very close to CO_2_ molecules [O-C-O···H-O- and HO···O-C-O at 2.11(6) and 2.63(3) Å, respectively]. Despite the supposed flexibility and structural adaptability that serine residues could offer, no important differences in the alcoholic chain conformations have been observed in the CO_2_@1 adsorbate with respect to 1, where only Zn-O_ser_ [ranging from 1.935(3) to 2.191(4) Å] distances show a slight contraction with respect to 1, while Zn-O_water_ [1.992(4) Å], and Zn-N bond lengths [1.959(4) Å] fall in the range of the expected values for Zn(II) metal ions [47].

What is worth underlining is the unusual penta-coordination of Zn(II) ions observed in 1 and CO_2_@1, reminiscent (in geometry) of that observed for the catalytic metal ions of the di-zinc aminopeptidase from Aeromonas proteolytica (AAP) [48]. Surveys of the Cambridge Structural Database (CSD) show zinc ion coordination number frequencies of ca. 60% and 25% for 4 and 6 coordination numbers, respectively. Interestingly, the zinc ion coordination prevalence in protein sites depends on whether the zinc plays a structural or a catalytic role. In structural zinc sites, the occurrence rate for 4, 5, and 6 coordination numbers is 80%, 6%, and 12%, respectively; whereas in catalytic zinc sites, the occurrence rate for 4, 5, and 6 coordination is 47%, 45%, and 6%, respectively [49]. Thus, five coordinate, or geometrically strained zinc sites, may represent sites equipped for catalysis, whereas four coordinate ideal tetrahedral zinc sites may represent stable sites affiliated with structural support.

Single-crystal X-ray experiment on samples of 1 after irradiation with UV light (without BG dye) do not show the presence of CO_2_ molecules, just coordinated water and small voids with light diffuse electron density. This ruled out the possibility of CO_2_ adsorption from air or solvent in 1, and reinforced our hypothesis that CO_2_@1 comes from the decomposition of the BG dye.

**Figure 5:**
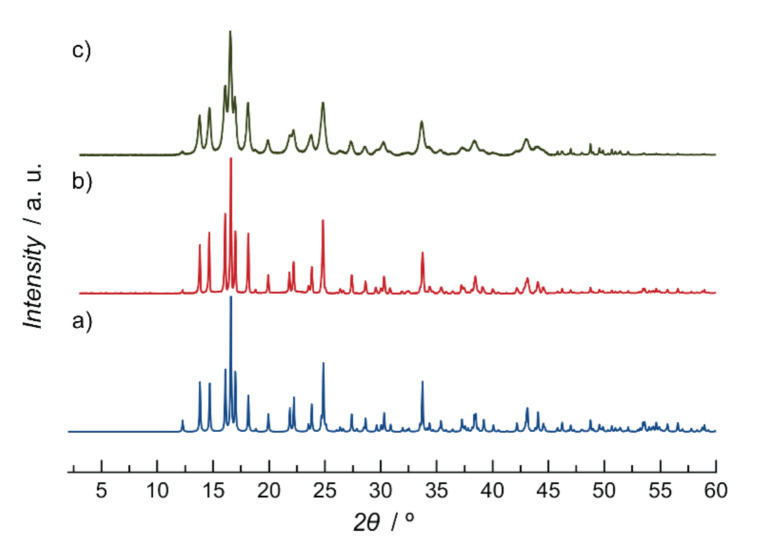
Calculated (**a**) and experimental (**b**) PXRD patterns of 1 in the 2θ range 2.0–60.0° at R.T. (**c**) Experimental PXRD pattern of 1 after three consecutive cycles of photocatalytic degradation of BG.

The experimental powder X-ray diffraction (PXRD) pattern of a polycrystalline sample of 1 is shown in Figure 5. It is consistent with the theoretical one and confirms the purity and homogeneity of the bulk sample. The solvent contents were determined by thermogravimetric analysis (TGA) under a dry N_2_ atmosphere (see Figure S7, Supporting Information) and helped to established the final chemical formula. Attempts to activate 1 (under different protocols) for measuring N_2_ isotherms proved unsuccessful, most likely related to its loss of structural stability when all molecule solvents were removed.

**Figure 6:**
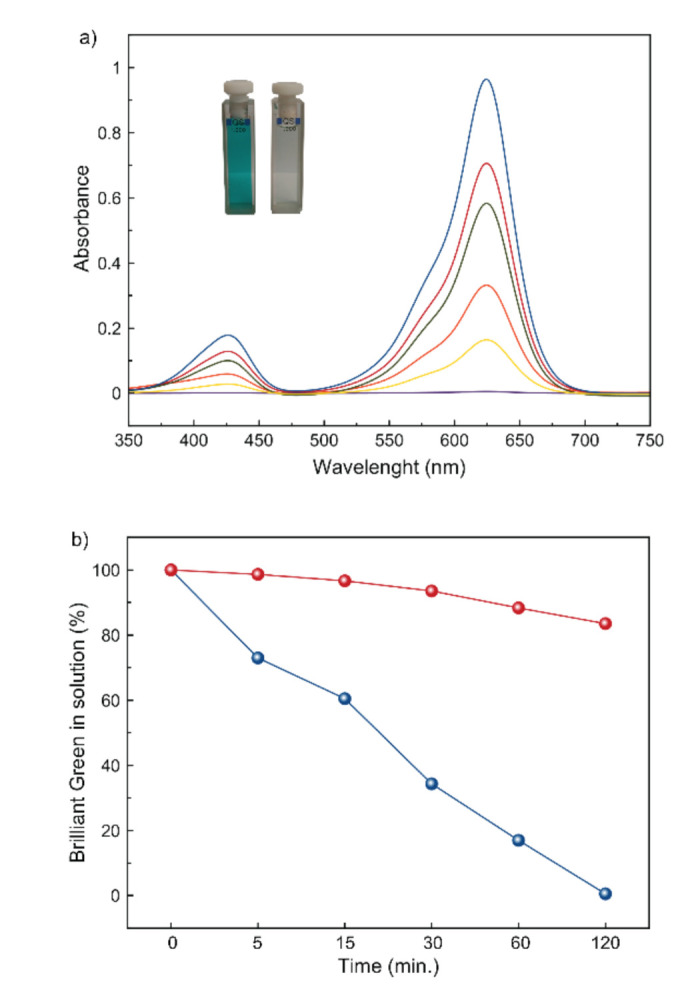
(**a**) Evolution with time of the UV-Vis absorption spectra of 10 ppm solutions of brilliant green in water in the presence of 25 mg of a polycrystalline sample of 1. Blue: *t* = 0; Red: *t* = 5 min.; Green: *t* = 15 min.; Orange = 30 min.; Yellow: *t* = 60 min.; Purple: *t* = 120 min. The inset shows the BG solutions at t = 0 min. (left) and 120 min. (right) of exposure of the BG solution with MOF 1. (**b**) Kinetic profile of the degradation of brilliant green under irradiation at 250 nm in the presence of MOF 1 (blue) and with no photocatalyst (red).

The photocatalytic activity of MOF 1 for the degradation of BG dye was then investigated. For this purpose, 25 mg of 1 were suspended in 50 mL of a 10 ppm aqueous solution of BG. Prior to irradiation, the mixture was kept in the dark for 30 min to verify that degradation only occurs under irradiation. After that period, the suspension started to be irradiated, under mild stirring at 250 nm. At different times (5, 15, 30, 60, and 120 min), 1 mL aliquots were taken, centrifuged, and diluted, and their UV–Vis absorption spectra were registered (Figure 6a). An identical experiment was performed, under the same conditions, in the absence of 1 (Figure S8). In order to have a better characterization of 1, we performed UV-Vis diffuse reflectance spectroscopy, which revealed a strong adsorption band below 350 nm (Figure S9).

Figure 6 shows the photodegradation efficiency of 1 towards BG. Such efficiency was evaluated by measuring the decrease in the characteristic absorption bands of BG dye, which appear at 420 nm and 625 nm, respectively. Thus, under irradiation in the presence of 1, it a gradual decrease of both peaks with time can be observed, until the vanish completely after 120 min (Figure 6a), which indicates that 100% of BG dye is eliminated after that time (Figure 6b). In turn, the same experiment, in the absence of 1, (Figure S8 and Figure 6b), shows a very smooth decrease of the UV–Vis absorption bands, confirming the key role of 1 as photocatalyst. Tauc plot of the Kubelka–Munk function (Figure S10) allowed us to obtain an estimation of the optical band of 1 (3.03 eV), which evidenced the suitability of it to perform the degradation of BG. Moreover, the reuse capability of 1 was stablished by both UV–Vis and PXRD experiments. On the one hand, two more UV–Vis vs. time experiments were carried out, using 1 as photocatalyst, with identical results (Figure S11), confirming that 1 can be employed in at least 3 cycles. On the other hand, PXRD patterns of 1 after three cycles are identical to those of the synthesized material suggesting that no degradation occurs during the photocatalytic process (Figure 5c).

The full degradation of the organic dye was also supported by thermogravimetric analysis coupled with mass spectrometry (TGA-MS) on CO_2_@1 (Figure S12). The weight loss in TGA led to two peaks in MS spectra with the mass to charge ratio (*m*/*z*) of 18 and 44, which can be attributed to water and carbon dioxide. However, the amount of CO_2_ desorbed was lower than that obtained from X-ray crystallography. Most likely, this is related to some losses of CO_2_ during the handling of the measurement itself. The larger size of brilliant green dye compared to 1 pore size precluded the adsorption of the organic dye on MOF channels to perform the photocatalytic event. Thus, the photocatalytic activity of 1 most likely will arise from the crystal surface. With the aim of confirming the photodegradation’s occurrance at the surface of 1, we measured the evolution of the BG concentration in solution when it is put in contact with 1 during 30 min in the dark. As can be observed in Figure S13, there is no appreciable difference in the BG concentration in solution, which supports our hypothesis. A leaching test ruled out the possibility of decomposition products of Zn-based MOF being responsible for the photocatalytic degradation of brilliant green. This was also supported, in an indirect manner, by the maintenance of both the activity in the recyclability studies (Figure S11) and the high crystallinity of PXRD diffraction patterns after the photocatalytic process (Figure S5c).

A plausible mechanism for the degradation of BG dye is shown in Scheme S1. With the aid of UVC light, 1 generates electron and hole pairs. While the electrons jump to the conducting band, the holes remain in the valence band. The holes were scavenged by water molecules leading to energetically reactive hydroxyl radicals, and the photogenerated electrons react with O_2_ to produce the superoxide radical anions of oxygen. From them, based on literature precedents [50,51,52,53], the OH• is believed to be the dominant oxidizing agent for the mineralization of BG dye into CO_2_ and H_2_O. ^1^H NMR of solutions after photocatalytic experiment do not show any peaks (apart from H_2_O) that can be attributed to any known intermediate from a previously reported study.^55^ With the aim of exploring the formation of intermediate species, we performed liquid chromatography/tandem mass spectrometry (LC-MS/MS) on solutions after 30 min. of UV irradiation, where we were able to identify four degradation molecules (Scheme S2) [54].

## 3. Conclusions

In summary, we have reported a novel, eco-friendly Zn-based MOF with the formula {Zn_2_[(*S,S*)-serimox](H_2_O)_2_] ··· H_2_O (1) and its performance as a photocatalyst of BG aqueous solutions. This MOF with good water stability was able to efficiently photodegrade 10 ppm aqueous solutions of BG in 120 min. Indeed, UV-Vis spectroscopy measurements of irradiated solutions, with and without the presence of 1, clearly revealed the photodegradation role of the framework. Single-crystal X-ray crystallography was applied not only to structurally characterize the pristine structure of 1, but more interestingly, after the photocatalytic process. The resolution of the crystal structure of CO_2_@1 allowed us to confirm the total degradation of the dye and the presence of CO_2_ molecules (from the degradation process) retained within the irregular hydrophilic channels of 1. Reusability tests of 1, with up to 3 cycles of the photodegradation process, evidenced structural and performance robustness, which further confirms the viability of 1 as an efficient photocatalyst. Our current work is focused on extending this study to other Zn-based MOFs derived from natural amino acids.

## Data Availability

CCDC 2062289-2062290 contains the supplementary crystallographic data for this paper. These data can be obtained free of charge via www.ccdc.cam.ac.uk/data_request/cif (accessed on 3 July 2021), or by emailing data_request@ccdc.cam.ac.uk, or by contacting The Cambridge Crystallographic Data Centre, 12 Union Road, Cambridge CB2 1EZ, UK; Fax: +44-1223-336033.

## Supplementary Materials

The following are available online at, experimental preparation, analytical and spectroscopic characterization of 1, and additional Figures S1–S13, Table S1, Schemes S1 and S2.

## Sample Availability

Samples of compounds 1 is available from the authors.
