# Supplementary material for: Photodegradation of Brilliant Green Dye by a Zinc bioMOF and Crystallographic Visualization of Resulting CO2
Source: Molecules. 2021 Jul 5;26(13):4098. doi: 10.3390/molecules26134098 (PMC8272194; doi:10.3390/molecules26134098)
Supplement: Supplementary file 1 [file molecules-26-04098-s001.zip › molecules-1275757-supplementary.pdf]

Electronic Supporting Information (ESI) for the manuscript:

Photodegradation of Brilliant Green Dye by a Zinc bioMOF and Crystallographic Visualization of resulting CO_2

Paula Escamilla, Marta Viciano-Chumillas, Rosaria Bruno, Donatella Armentano
Emilio Pardo and Jesús Ferrando-Soria

Experimental Section

Materials. All chemicals were of reagent grade quality. They were purchased from commercial sources and used as received. The proligand H_2Me_2-(*S*,*S*)-serimox was prepared as previously reported.[1]

$(Me_4N)_2\{Ni_2[(S,S)\text{-serimox}](OH)_2\} \cdot 5H_2O$. An aqueous solution (100 mL) of H_2Me_2-(*S*,*S*)-serimox (5.00 g, 17 mmol) was treated with a 25% methanolic solution of Me_4NOH (28 mL, 68 mmol). Then, to the resulting solution, $NiCl_2 \cdot 6H_2O$ (4.43 g, 34 mmol) in 30 mL of water was added dropwise under magnetic stirring. The green solution was filtered to remove the solid precipitate and concentrated in a rotatory evaporator to afford a green solid that was gently washed with acetone filtered off and dried under vacuum. Yield: 7.97 g, 83%. IR (KBr): ν = 3389 cm^{-1} (O−H), 3019 and 2961 cm^{-1} (C−H), 1604 cm^{-1} (C=O).

$\{Zn^{II}_2[(S,S)\text{-serimox}](H_2O)_2] \cdot H_2O$ (1). Two synthetic procedures can be followed to obtain **1**. In a direct one, an aqueous solution (30 mL) of H_2Me_2-(*S*,*S*)-serimox (3.6 g, 12 mmol) and a 25% methanolic solution of Me_4NOH (19.8 mL, 48 mmol) was added dropwise to an aqueous solution $Zn(NO_3)_2 \cdot 6H_2O$ (7.15 g, 26 mmol) in 5 mL. The solution mixture was stirred overnight and a white powder was obtained and collected by filtration, washed with water and methanol. Yield: 4.7 g, 88%. Anal.: calcd for $C_8H_{14}N_2O_{11}Zn_2$ (444.96): C, 21.59; H, 3.17; N, 6.30%. Found: C, 21.83; H, 3.23; N, 6.42%. IR (KBr): ν = 1604 cm^{-1} (C=O).

Alternatively, it could be followed an indirect pathway. For that, $(Me_4N)_2\{Ni_2[(S,S)\text{-serimox}](OH)_2\} \cdot 5H_2O$ (1.37 g, 2.44 mmol) was dissolved in 10 mL mixture water/methanol (1:1) and then, $Zn(NO_3)_2 \cdot 6H_2O$ (1.45 g, 4.88 mmol) was added under stirring. After further stirring for 10 h, at room temperature, a white powder was obtained and collected by filtration, washed with water and methanol. Yield: 0.91 g, 83%;

Anal.: calcd for $C_8H_{14}N_2O_{11}Zn_2$ (444.96): C, 21.59; H, 3.17; N, 6.30%. Found: C, 20.99; H, 3.14; N, 6.09%. IR (KBr): ν = 1604 cm^{-1} (C=O).

Suitable single crystals of **1** for X-ray structural analysis were obtained by slow diffusion, in an H-shaped tube, of H_2O/MeOH (1:1) solutions containing stoichiometric amounts of $(Me_4N)_2\{Ni_2[(S,S)\text{-serimox}](OH)_2\}\cdot 5H_2O$ (0.13 g, 0.18 mmol) in one arm and $Zn(NO_3)_2\cdot 6H_2O$ (0.072 g, 0.36 mmol) in the other. They were isolated by filtration on paper and air-dried.

$\{Zn^{II}_2[(S,S)\text{-serimox}](H_2O)_2]\cdot CO_2$ (CO_2@1).

Suitable single crystals of **CO_2@1** for X-ray structural analysis were obtained by irradiating crystals of **1** with an UV lamp in the range of λ = 250-350 nm, for 60 min and then left crystals in a sealed glass tube containing aqueous solution of BG dye for one week.

Physical Techniques. Elemental (C, H, S, N) analyses were performed at the Microanalytical Service of the Universitat de València. FT–IR spectra were recorded on a Perkin-Elmer 882 spectrophotometer as KBr pellets. The thermogravimetric analyses were performed on crystalline samples under a dry N_2 atmosphere with a Mettler Toledo TGA/STDA 851e thermobalance operating at a heating rate of 10 ºC min^{-1}. Thermogravimetric analysis coupled with mass spectrometry was performed at the Servicios Técnicos de Investigación of the Universidad de Alicante.

X–ray Powder Diffraction Measurements. Polycrystalline samples of **1** –before and after photocatalytic experiments– were introduced into 0.5 mm borosilicate capillaries prior to being mounted and aligned on an Empyrean PANalytical powder diffractometer, using Cu Kα radiation (λ = 1.54056 Å). For each sample, five repeated

measurements were collected at room temperature (2θ = 2–60°) and merged in a single diffractogram.

X-ray crystallographic data collection and structure refinement. Crystals of **1**, and **CO_2@1** (crystals of **1** left in a sealed glass tube containing aqueous solution of BG dye for one week, after irradiation in the range 250-350 nm for 60 min) were selected and mounted on a MiTeGen MicroMount in Paratone oil and very quickly placed on a liquid nitrogen stream cooled at 100 K, to avoid the possible degradation upon exposure to air. Diffraction data were collected on a Bruker-Nonius X8APEXII CCD area detector diffractometer using graphite-monochromated Mo-K_α radiation (λ = 0.71073 Å). The data were processed through SAINT[1] reduction and SADABS[2] multi-scan absorption software. The structures were solved with the SHELXS structure solution program, using the Patterson method. The model was refined with version 2018/3 of SHELXL against F^2 on all data by full-matrix least squares.[3]

In the refinement of crystal structures all non–hydrogen atoms of the network were refined anisotropically. In **CO_2@1**, the C-C and C-O bond lengths of serine moieties have been restrained. It is reasonable and related to flexibility of aminoacid chains confined within pores, which are dynamic components of the frameworks. Restrains on bond lengths and angles of CO_2 molecules, to make the refinement more efficient, have been also applied.

All the hydrogen atoms of the network were set in calculated position and refined isotropically using the riding model. Hydrogen atoms on water molecules were found form ΔF map and refined with restrains on O-H length and H-O-H angle. In crystal structure of **1**, lattice water molecules, detected by TGA analysis, were not found by density map. It is likely due to dynamic disorder, as suggested by light residual diffuse densities. CO_2 molecules reside on special positions. They exhibit large thermal disorder

as expected and have been refined considering a population of 50% of the cells (0.5 as occupancy factor). Furthermore, atomic displacement parameters in carbon and oxygen atoms of CO_2 molecules expected to have essentially similar ADPs, have been restrained using EADP instruction.

A summary of the crystallographic data and structure refinement for the two compounds is given in Table S1. The comments for the alerts of level A and B (in **CO_2@1**, related to thermal and statistic disorder of CO2 molecules) are described in the CIFs using the validation reply form (vrf). CCDC reference numbers are 2062289-2062290 for **1** and **CO2@1**, respectively.

The final geometrical calculations and the graphical manipulations were carried out with PLATON[4] implemented in WinGX,[5] and CRYSTAL MAKER[6] programs, respectively.

Photocatalytic experiments

Photoirradiation experiments were performed inside a commercial photoreactor (LuzChem LZC-4V) with 14 UVC (250 nm) lamps. Absorption spectra were recorded on a Jasco V-670.

Table S1. Summary of Crystallographic Data for {Zn^{II}_2[(*S*,*S*)-serimox](H_2O)$_2$] · H_2O (1) and {Zn^{II}_2[(*S*,*S*)-serimox](H_2O)$_2$] · CO_2 (CO_2@1).

Compound	**1**	**CO_2@1**
Formula	$C_8Zn_2H_{12}N_2O_{10}$	$C_{8.5}Zn_2H_{12}N_2O_{11}$
M (g mol^{-1})	426.94	448.94
λ (Å)	0.71073	0.71073
Crystal system	Tetragonal	Tetragonal
Space group	$P4_12_12$	$P4_12_12$
a (Å)	7.6380(10)	7.5543(3)
c (Å)	24.390(4)	24.0962(12)
V (Å^3)	1422.9(4)	1375.11(12)
Z	4	4
ρ_{calc} (g cm^{-3})	1.993	2.169
μ (mm^{-1})	3.425	3.555
T (K)	100	100
θ range for data collection (°)	3.147 to 26.675	2.826 to 26.987
Completeness to θ = 25.0	100%	100%
Measured reflections	20277	4650
Unique reflections (Rint)	1507 (0.0672)	1449 (0.0301)
Observed reflections [$I > 2\sigma(I)$]	1319	1360
Goof	1.003	1.107
R^a [$I > 2\sigma(I)$] (all data)	0.0311 (0.0401)	0.0293 (0.0315)
wR^b [$I > 2\sigma(I)$] (all data)	0.0808 (0. 0.0855)	0.0845 (0.0837)
Flack parameter	0.00(1)	0.00(1)
CCDC	2062289	2062290

^{a} $R = \sum(|F_o| - |F_c|)/\sum|F_o|$. ^{b} $wR = [\sum w(|F_o| - |F_c|)^2/\sum w|F_o|^2]^{1/2}$.

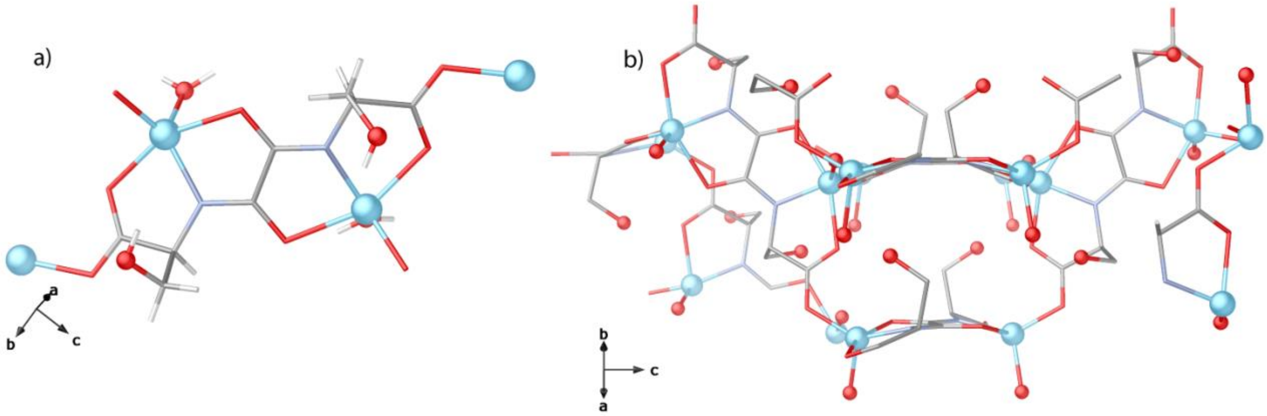

Fig. S1. Perspective views of SBUs a) and their assembly by means the carboxylate groups b) in **1**. Colour code: Zn atoms are represented as cyan spheres, C, N, H and O atoms from serimox ligands –with the exception of OH of serine residues– are represented as grey, cyan, white and red sticks. Oxygen atoms from -OH groups are represented as red spheres.

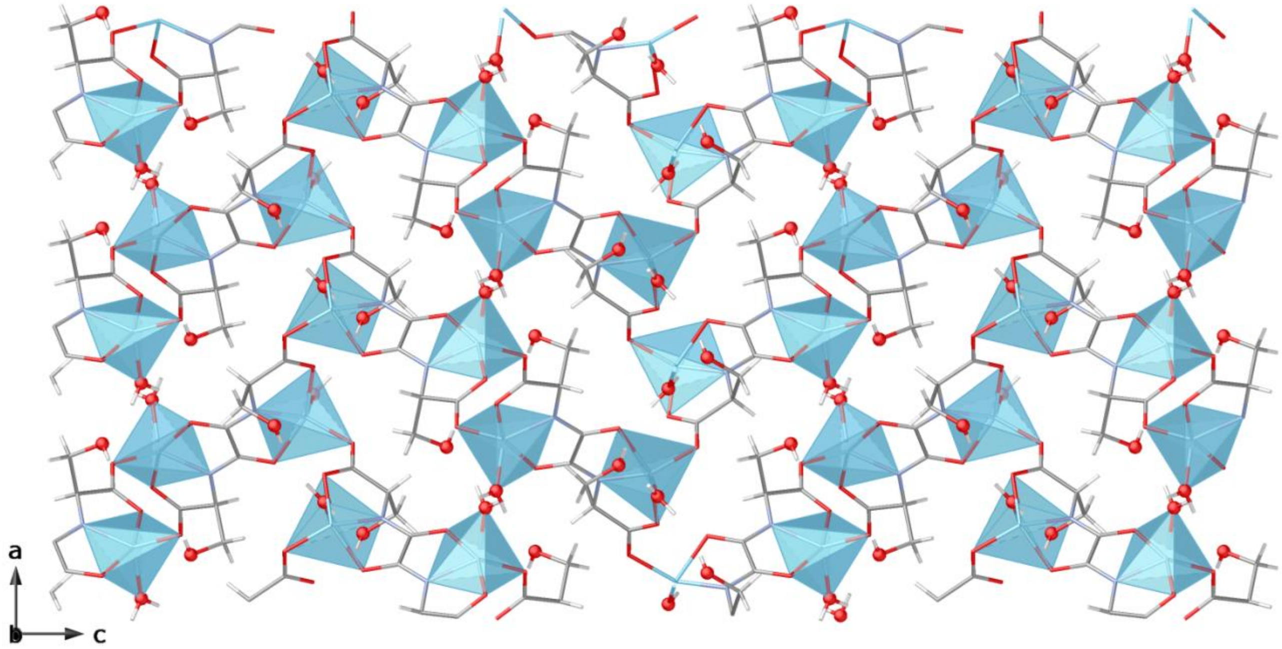

Fig. S2. View of the network of **1** along the *b* axes. Colour code: Zn atoms are represented as cyan polyhedra, C, N, H and O atoms from serimox ligands –with the exception of OH of serine residues– are represented as grey, cyan, white and red sticks. Oxygen atoms from -OH groups are represented as red spheres.

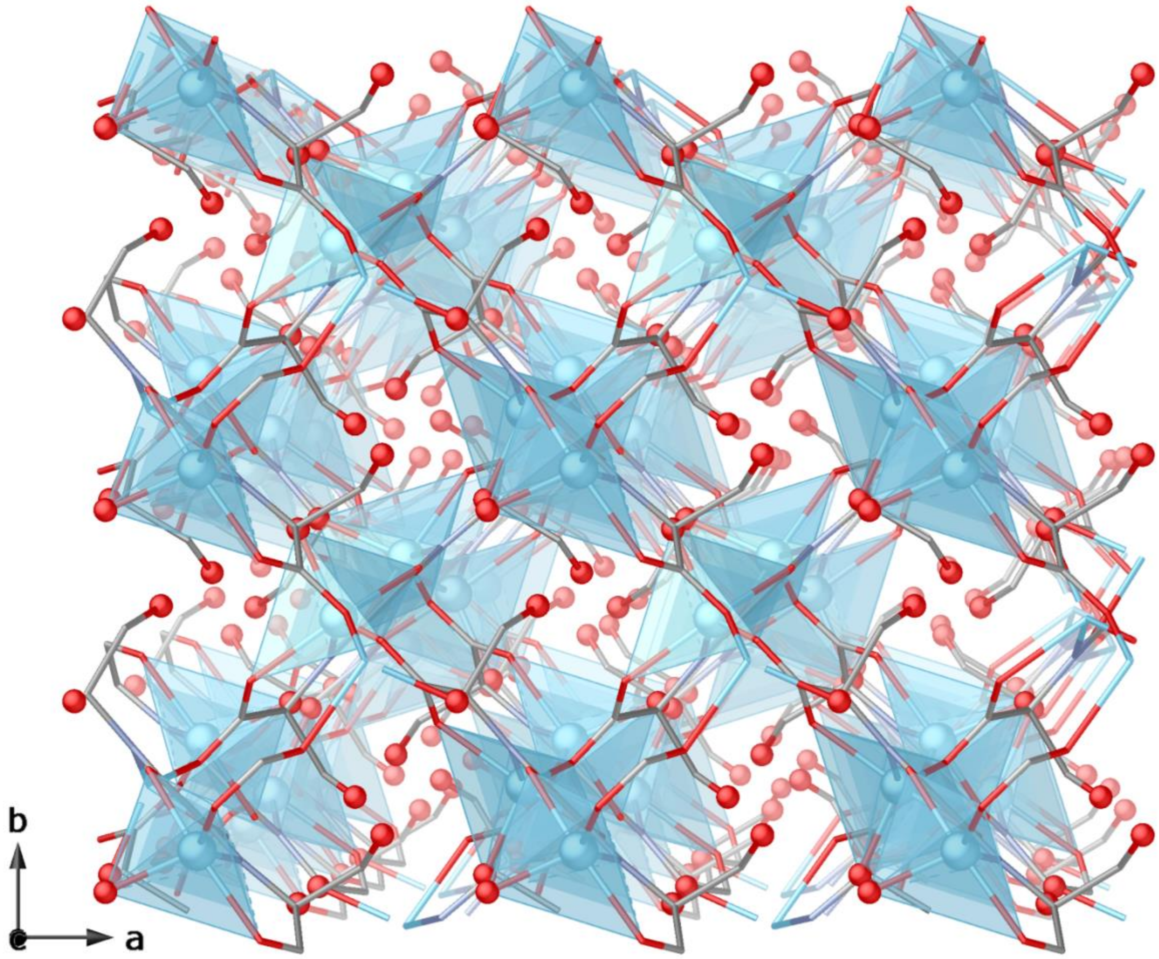

Fig. S3. Perspective view of the network of **1** along the *c* axes. Colour code: Zn atoms are represented as cyan polyhedra, C, N, H and O atoms from serimox ligands –with the exception of OH of serine residues– are represented as grey, cyan, white and red sticks. Oxygen atoms from -OH groups are represented as red spheres.

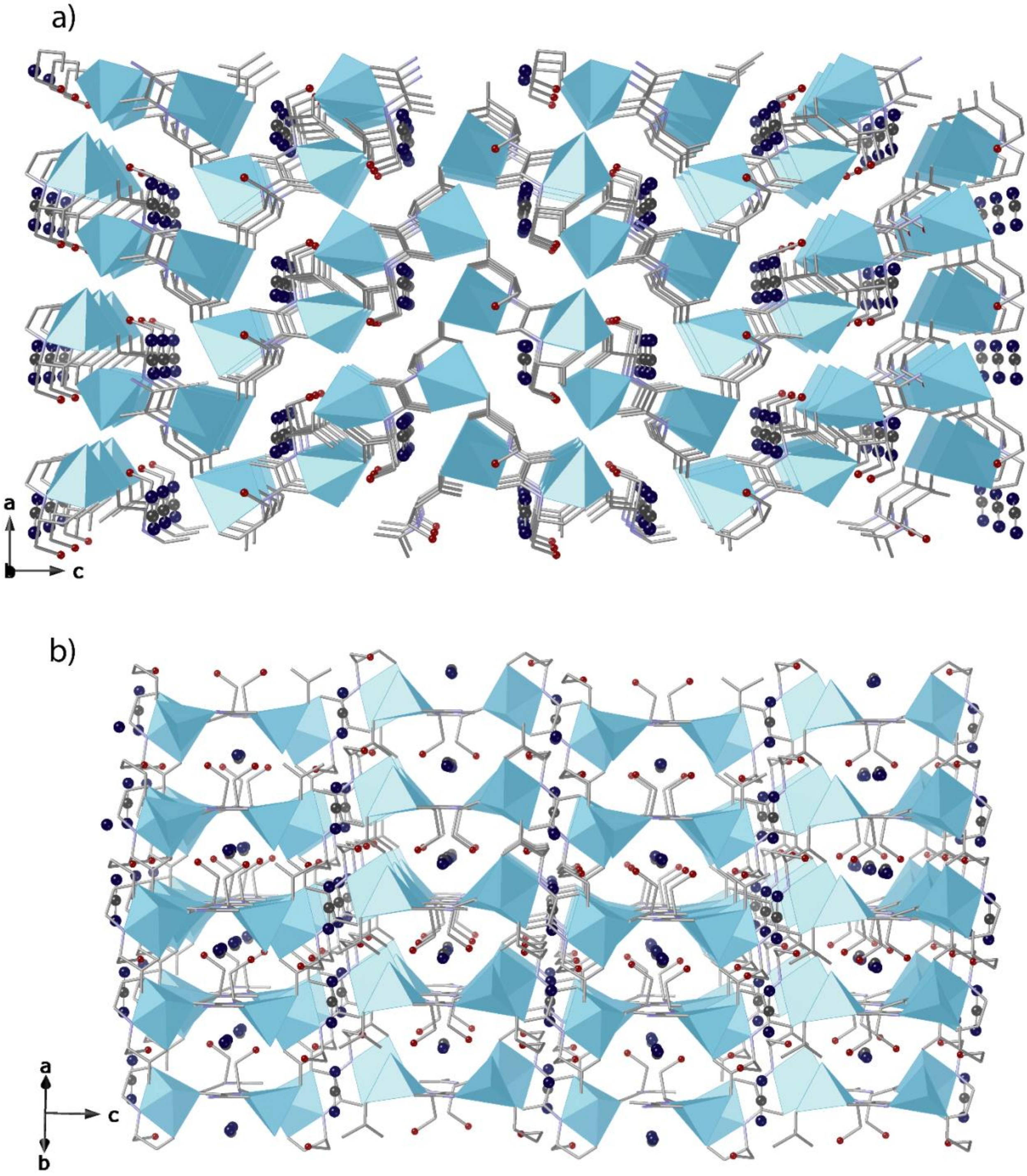

Fig. S4. Perspective views of the network of $\mathbf{CO_2@1}$ along the *b* and [1 1 0] directions (a and b, respectively) unveiling CO_2 molecules retained within voids. Colour code: Zn atoms are represented as cyan polyhedra, C, N, and O atoms from serimox ligands –with the exception of OH of serine residues– are represented as grey sticks. Oxygen atoms from -OH groups are represented as red spheres whereas oxygen atoms from guest molecules are represented as deep blue spheres. Hydrogen atoms have been omitted for the sake of clarity.

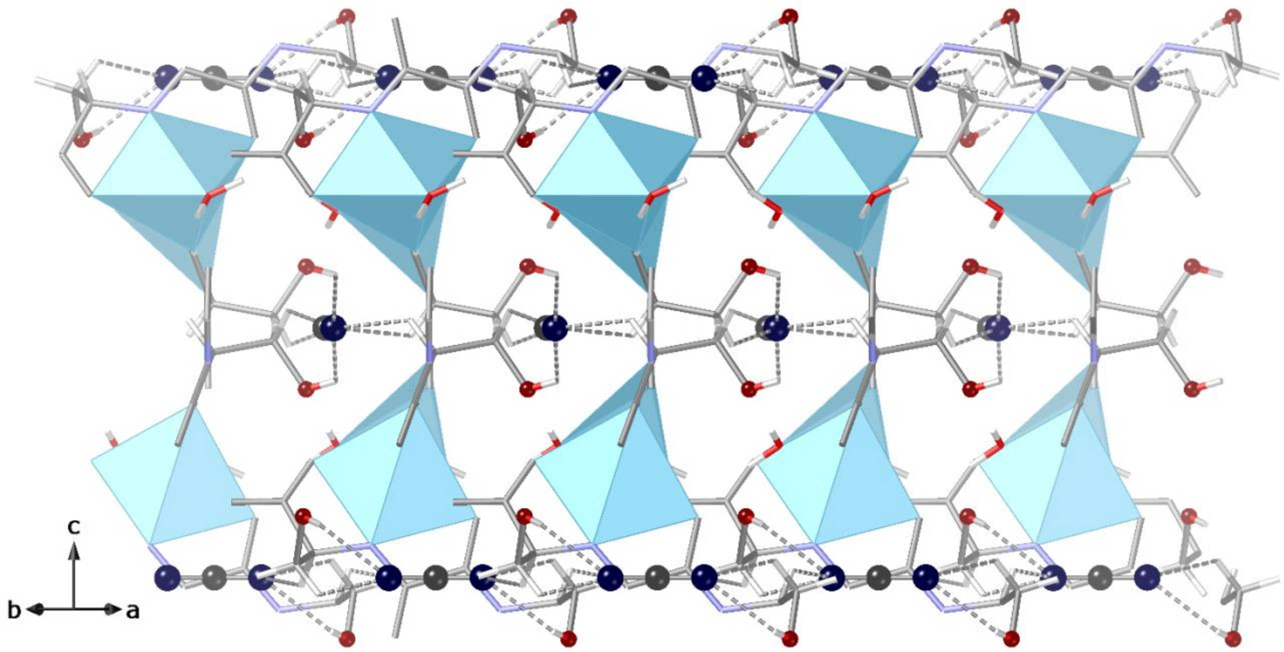

Fig. S5. Details of host-guest interactions in **CO_2@1** along the *c* [1 1 0] direction. Zn atoms are represented as cyan polyhedra, C, N, and O atoms from serimox ligands –with the exception of OH of serine residues– are represented as grey sticks. Oxygen atoms from -OH groups are represented as red spheres whereas oxygen atoms from guest molecules are represented as deep blue spheres.

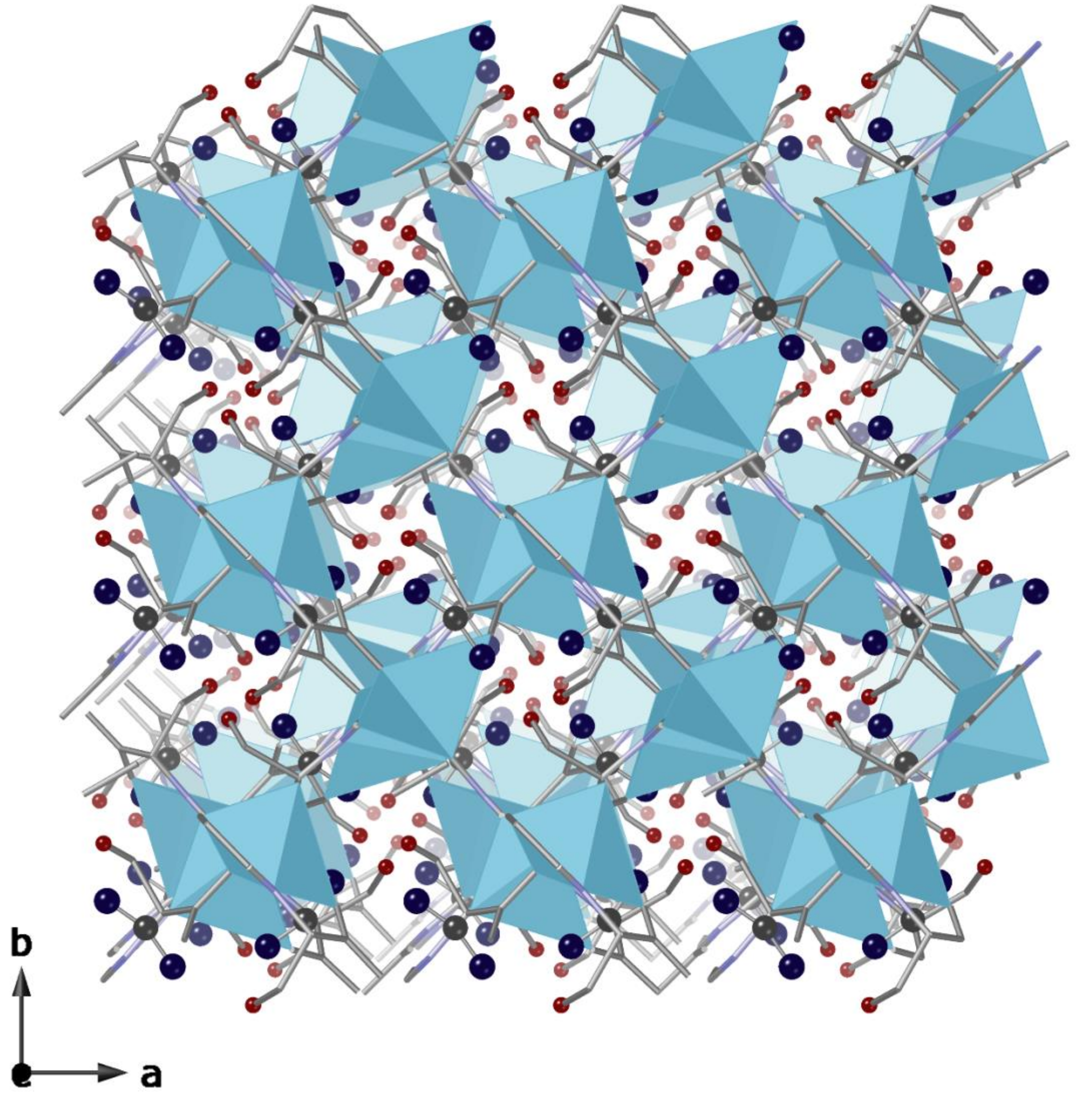

Fig. S6. Perspective view of the network of **CO_2@1** along the *c* direction unveiling CO_2 molecules retained within voids. Colour code: Zn atoms are represented as cyan polyhedra, C, N, and O atoms from serimox ligands –with the exception of OH of serine residues– are represented as grey sticks. Oxygen atoms from -OH groups are represented as red spheres whereas oxygen atoms from guest molecules are represented as deep blue spheres. Hydrogen atoms have been omitted for the sake clarity.

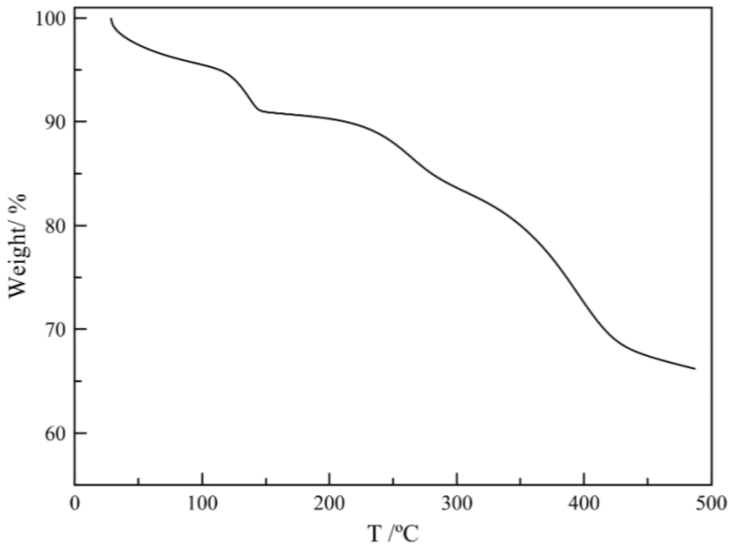

Fig. S7. Thermo-Gravimetric Analysis (TGA) of **1** under dry N_2 atmosphere.

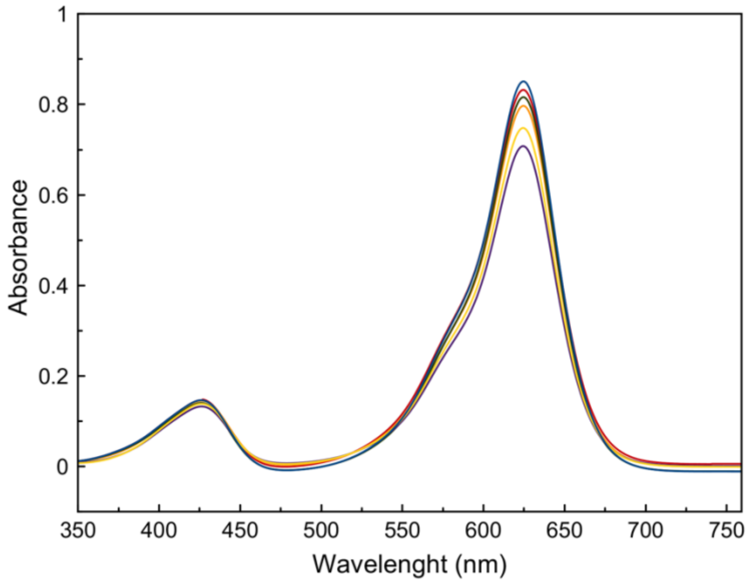

Fig. S8. Evolution with time of the UV-Vis absorption spectra of 10 ppm solutions of Brilliant green in water under irradiation at 250 nm. Blue: t = 0; Red: t = 5 min.; Green: t = 15 min.; Orange = 30 min.; Yellow: t = 60 min.; Purple: t = 120 min.

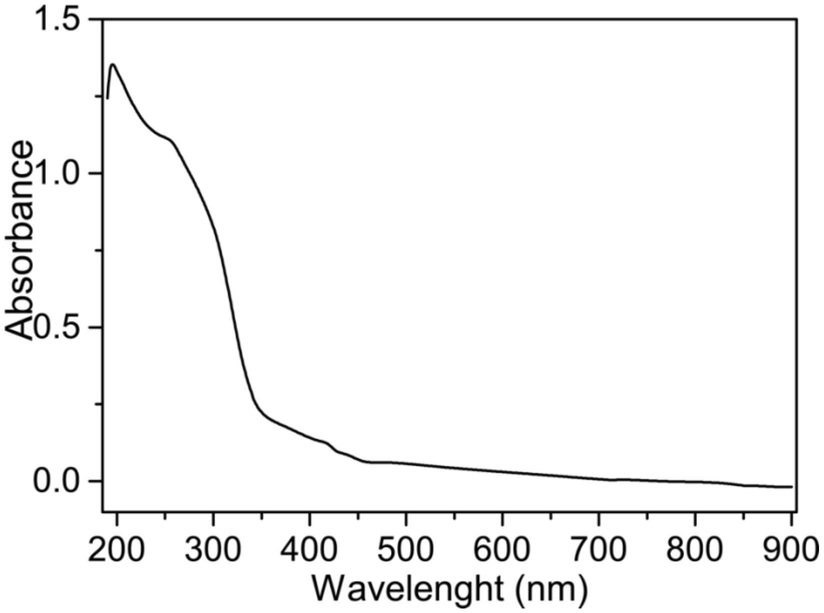

Fig. S9. UV-Vis spectrum of a polycrystalline sample of **1**.

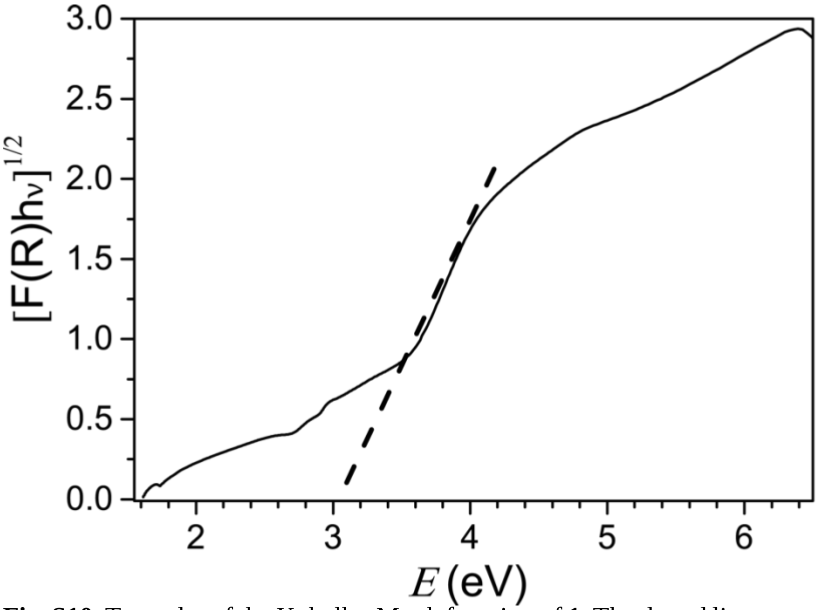

Fig. S10. Tauc plot of the Kubelka-Munk function of **1**. The dotted line corresponds to the regression fitting of the linear part of the plot. The optical band-gap of **1** was 3.03 eV.

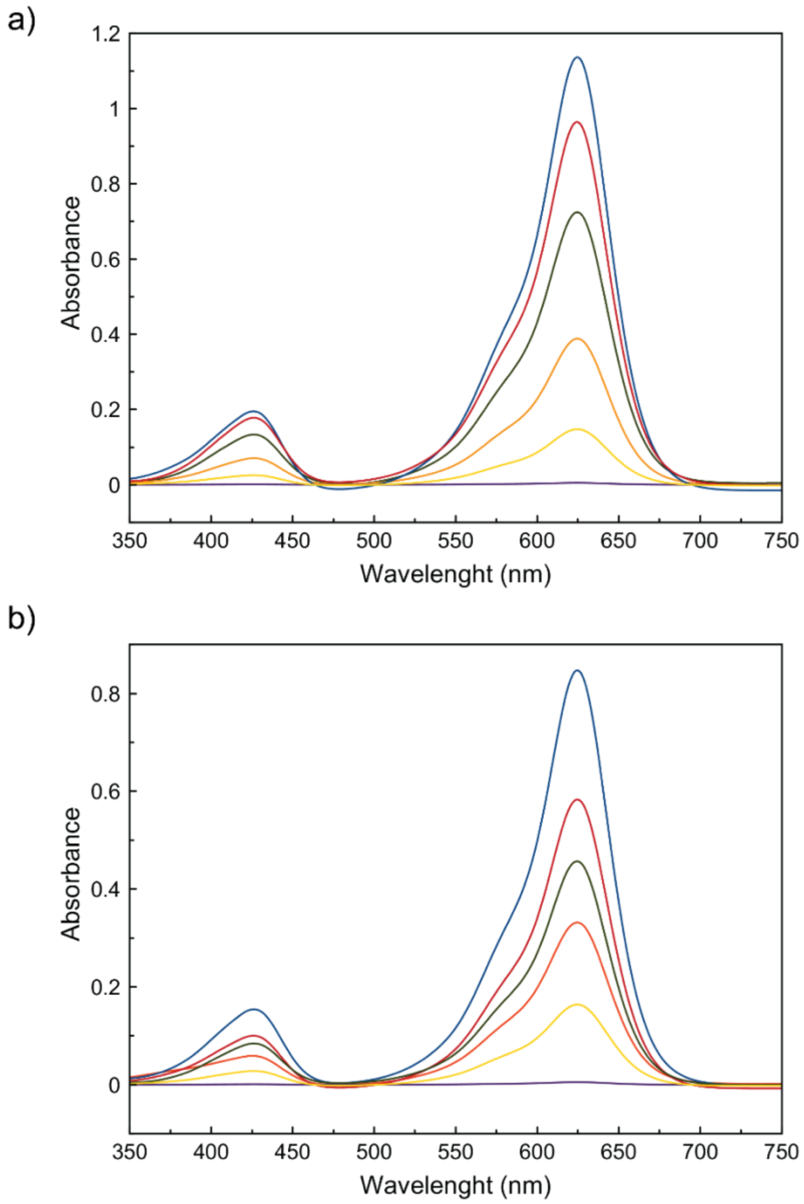

Fig. S11. Second (a) and third (b) cycle of the evolution with time of the UV-Vis absorption spectra of 10 ppm solutions of Brilliant green in water in the presence of 25 mg of a polycrystalline sample of **1**. Blue: *t* = 0; Red: *t* = 5 min.; Green: *t* = 15 min.; Orange = 30 min.; Yellow: *t* = 60 min.; Purple: *t* = 120 min.

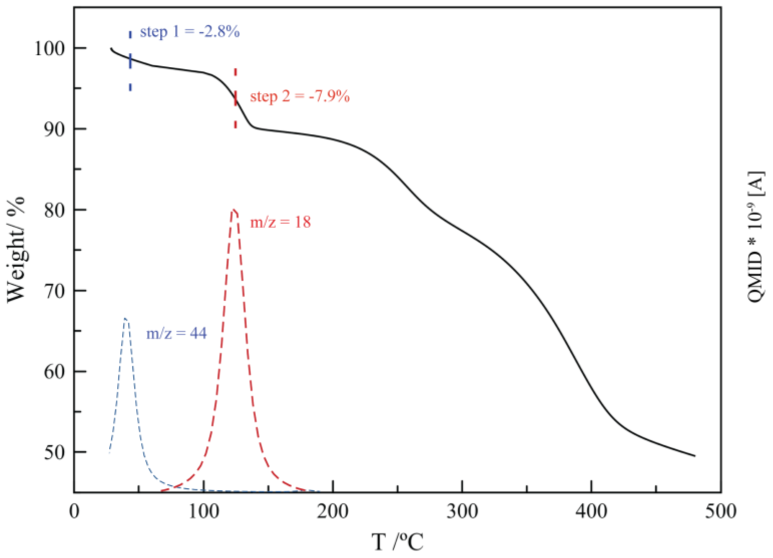

Fig. S12. Thermo-Gravimetric coupled with Mass Spectrometry (TG-MS) of **CO@1**. The emission of CO_2 (*m*/*z* = 44) and water (*m*/*z* = 18) was monitored with mass spectrometry.

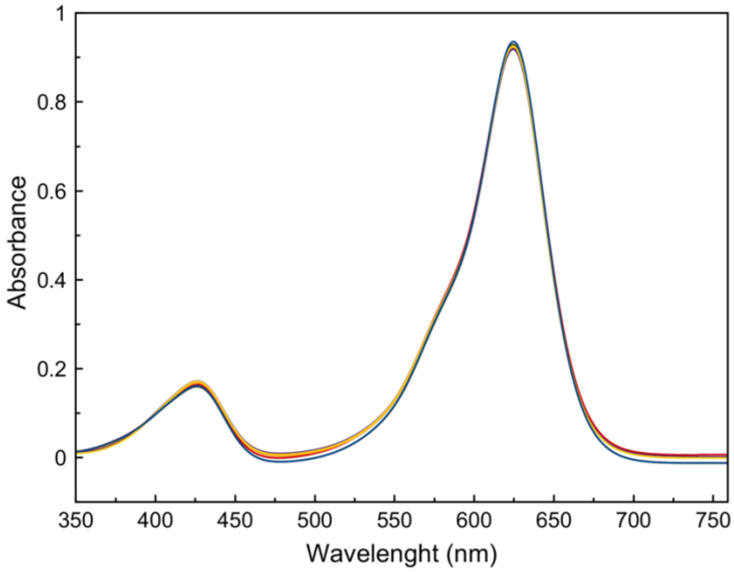

Fig. S13. Evolution with time of the UV-Vis absorption spectra of 10 ppm solutions of Brilliant green in water in the dark. Blue: *t* = 0; Red: *t* = 5 min.; Green: *t* = 10 min.; Orange = 20 min.; Yellow: *t* = 25 min.; Purple: *t* = 30 min..

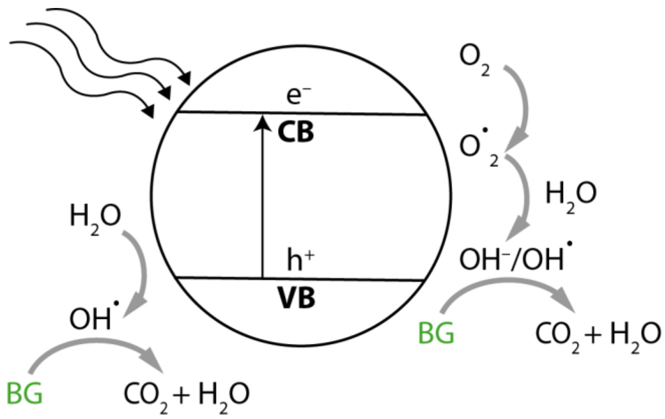

Scheme. S1. Plausible photocatalytic mechanism of BG dye degradation by **1**.

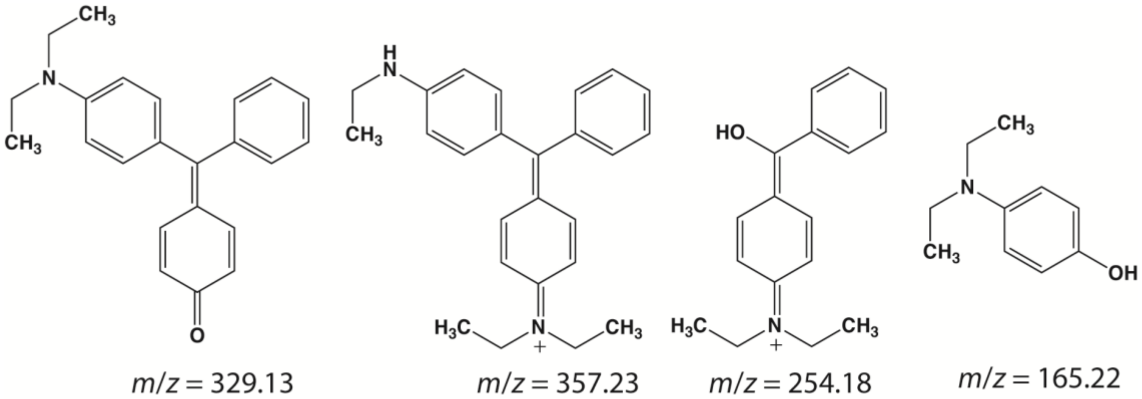

Scheme. S2. Identified species by liquid chromatography/tandem mass spectrometry after 30 min of photocatalytic experiment.

References

1. Mon, M.; Bruno, R.; Ferrando-Soria, J.; Bartella, L.; Di Donna, L.; Talia, M.; Lappano, R.; Maggiolini, M.; Armenatno, D.; Pardo, E. Crystallographic snapshots of host-guest interactions in drugs@metal-organic frameworks: towards mimicking molecular recognition processes. *Mater. Horiz.* **5**, 683 (2018).
2. SAINT, version 6.45, Bruker Analytical X-ray Systems, Madison, WI, 2003.
3. Sheldrick G.M. SADABS Program for Absorption Correction, version 2.10, Analytical X-ray Systems, Madison, WI, 2003.
4. (a) Sheldrick, G. M. Crystal structure refinement with SHELXL. *Acta Cryst. C* **71**, 3 (2015). (b) Sheldrick, G. M. A short history of SHELX. *Acta Cryst. A* **64**, 112 (2008). (c) SHELXTL-2013/4, Bruker Analytical X-ray Instruments, Madison, WI, 2013.
5. (a) Spek, A. L. *PLATON* SQUEEZE: a tool for the calculation of the disordered solvent contribution to the calculated structure factors. *Acta Crystallogr. Sect. C-Struct. Chem*. **71**, 9 (2015). (b) Spek, A. L. Structure validation in chemical crystallography. *Acta Crystallogr. Sect. D, Biol. Crystallogr.* **65**, 148 (2009).
6. Farrugia, L. J. *WinGX* suite for small-molecule single-crystal crystallography. *J. Appl. Crystallogr.* **32**, 837 (1999).
7. Palmer, D. CRYSTAL MAKER, Cambridge University Technical Services, C. No Title, 1996.
8. Kresse, G.; Furthmüller, Efficient iterative schemes for *ab initio* total-energy calculations using a plane-wave basis set, *J. Phys. Rev. B* **54**, 11169 (1996).
9. a) Perdew, J. P.; Burke, K.; Ernzerhof, M. Generalized Gradient Approximation Made Simple. *Phys. Rev. Lett.* **77**, 3865 (1996). b) Perdew, J. P.; Burke, K.; Ernzerhof, M. Generalized Gradient Approximation Made Simple. *Phys. Rev. Lett.* **78**, 1396 (1997).
10. Blöchl, P. E. Projector augmented-wave method. *Phys. Rev. B* **50**, 17953 (1994).
11. a) Henkelman, G.; Arnaldsson, A.; Jonsson, H. A fast and robust algorithm for Bader decomposition of charge density. *Comput. Mater. Sci.* **36**, 354 (2006). b) Sanville, E.; Kenny, S. D.; Smith, R.; Henkelman, G. Improved Grid-Based Algorithm for Bader Charge Allocation. *J. Comput. Chem.* **28**, 899 (2007).
12. Simonelli, L. et al. CLÆSS: The hard X-ray absorption beamline of the ALBA CELLS synchrotron. *Cog. Phys.* **3**, 1231987 (2016).
13. Ravel, B.; Newville, M. ATHENA, ARTEMIS, HEPHAESTUS: data analysis for X-ray absorption spectroscopy using IFEFFIT, *J. Synchr. Rad.* **12**, 537 (2005).
14. a) Pd-S: Grønvold, F.; Røst., E. The crystal structure of PdSe2 and PdS2. *Acta Cryst.* **10**, 329 (1957). b) Pd-Pd: Whyckoff R. W. G. Crystal Structures **1**, 7 (1963) Second edition. Interscience Publishers, New York, New York.
